# LightDock goes information-driven

**DOI:** 10.1093/bioinformatics/btz642

**Published:** 2019-08-16

**Authors:** Jorge Roel-Touris, Alexandre M J J Bonvin, Brian Jiménez-García

**Affiliations:** Bijvoet Center for Biomolecular Research, Faculty of Science, Department of Chemistry, Utrecht University, Utrecht, The Netherlands

## Abstract

**Motivation:**

The use of experimental information has been demonstrated to increase the success rate of computational macromolecular docking. Many methods use information to post-filter the simulation output while others drive the simulation based on experimental restraints, which can become problematic for more complex scenarios such as multiple binding interfaces.

**Results:**

We present a novel method for including interface information into protein docking simulations within the LightDock framework. Prior to the simulation, irrelevant regions from the receptor are excluded for sampling (filter of initial swarms) and initial ligand poses are pre-oriented based on ligand input information. We demonstrate the applicability of this approach on the new 55 cases of the Protein–Protein Docking Benchmark 5, using different amounts of information. Even with incomplete or incorrect information, a significant improvement in performance is obtained compared to blind *ab initio* docking.

**Availability and implementation:**

The software is supported and freely available from https://github.com/brianjimenez/lightdock and analysis data from https://github.com/brianjimenez/lightdock_bm5.

**Supplementary information:**

Supplementary data are available at *Bioinformatics* online.

## 1 Introduction

Computational tools are essential to predict and describe three-dimensional (3D) interactions between biomolecules. In particular, integrative approaches, i.e. data- or information-driven, are broadly used in order to combine experimental data with docking simulations (Jiménez-García *et al.*, 2013; Quignot *et al.*, 2018; Rodrigues and Bonvin, 2014; Russel *et al.*, 2012; De Vries *et al.*, 2010, 2015). In the context of molecular docking, there are still two main challenges: (i) searching the conformational space, especially in the case of highly flexible molecules, and (ii) evaluating and selecting near-native poses out of the generated conformers, which is usually referred to as scoring.

LightDock (Jiménez-García *et al.*, 2018) is a multiscale flexible framework for the 3D determination of binary protein complexes based on the Glowworm Swarm Optimization (GSO) (Krishnanand and Ghose, 2009) algorithm that systematically optimizes the generated docking poses towards those energetically more favourable at every simulation step.

Introducing restraints or biases in docking is a powerful mechanism to drive the simulation towards poses that satisfy those restraints (Dominguez *et al.*, 2003). Here we describe and benchmark an updated implementation of LightDock that now supports the use of information to drive or bias the docking simulation by filtering out swarms, pre-orienting ligand poses based on the available information and biasing the scoring energy upon satisfied residue contact restraints.

The results on the benchmark demonstrate a high performance of LightDock when used in combination with additional information. We also explore different scenarios with less accurate or incorrect information to show the versatility and robustness of our approach.

## 2 Materials and methods

Due to the nature of the LightDock framework, information about interfacial residues can be applied at different levels depending on the availability of information for the receptor, the ligand or both. On the receptor side, we filter out initial swarms that are not in the proximity of the defined restraints (Supplementary Material S1), with the collateral advantage of reducing considerably the computation time. On the ligand side, we orient initial poses based on randomly selected receptor-ligand restraint pairs (Supplementary Material S2). Steps S1 and S2 are only performed at the initial setup stage of the simulation.

Finally, we bias the scoring according to the percentage of satisfied residue contact restraints (Supplementary Material S3) at every simulation step. The biasing of specific residues could be disabled if they are defined as passive by the user (only S1 and S2 steps at the setup stage will therefore apply).

## 3 Results

The latest release of LightDock (0.7.0) (Jimenez *et al.*, 2019), which now supports the use of information to drive the docking in the format of residue restraints, was tested on the 55 unbound new entries of the Protein Docking Benchmark version 5 (Vreven *et al.*, 2015), which represents an unbiased dataset where no software/scoring functions were trained in, and includes 16 antibody-antigen complexes. We defined various scenarios to demonstrate its versatility and robustness as follows:

1. *TI*: True interface, defined as those residues at 3.9Å distance (as also defined in LIGPLOT (Wallace *et al.*, 1995) by default) from the partner molecule. This is an ideal case where a fully accurate definition of interface residues is available, but no specific contacts are defined.
2. *TI_50_*: We defined two different artificial interfaces with half of the *TI* residues and equal number of non-interfacial residues forming a contiguous patch as described in Supplementary Material S4. Results are reported as averaged success rates of both runs (using each of the designed interfaces).
3. *TI_25_*: In the same way as in *TI_50_*, we defined four different sets of restraints with one fourth of the original *TI* and three times more false positive residues forming a contiguous patch (Supplementary Material S4). Results are reported as averaged success rates of the four docking calculations (each one using a different artificially designed interface).
4. *TI_REC_*: Only the *TI* from the receptor is considered as restraints.
5. *TI_REC-50_*: As in *TI_50_*, but only considering the receptor interface residues.
6. *TI_REC-25_*: As in *TI_25_*, but only considering the receptor interface residues.
7. *TI_SINGLE_*: Only one receptor-ligand residue pair, making a real contact, is used as residue restraints.
8. *TI_ONE_*: Only one residue on the receptor, the same one as defined in TI_SINGLE_, is considered as restraint, without any information on the ligand side.

While several docking algorithms allow the use of information as *a posteriori* filter, LightDock incorporates this data *a priori*. If residue restraints are provided, irrelevant sampling regions are excluded by filtering the initial swarms and pre-orienting the initial poses (glowworms). This method not only represents a more efficient way as compared to post-docking approaches but also leads to a higher success rate. To test this hypothesis, we have filtered the *BLIND* predictions (*BLIND_filtered_*) according to an accurate description of the interface (residue restraints as used in *TI*). As shown in Supplementary Figure S2, post-filtering results in a clear improvement of the performance compared to *ab initio* docking. Nevertheless, when using this information prior the docking (*TI*), the success rate considerably increases reaching a maximum of 98.2% for the Top50 (54 of 55 cases) compared to a moderate 40% in BLIND_filtered_.

**Fig. 1.**
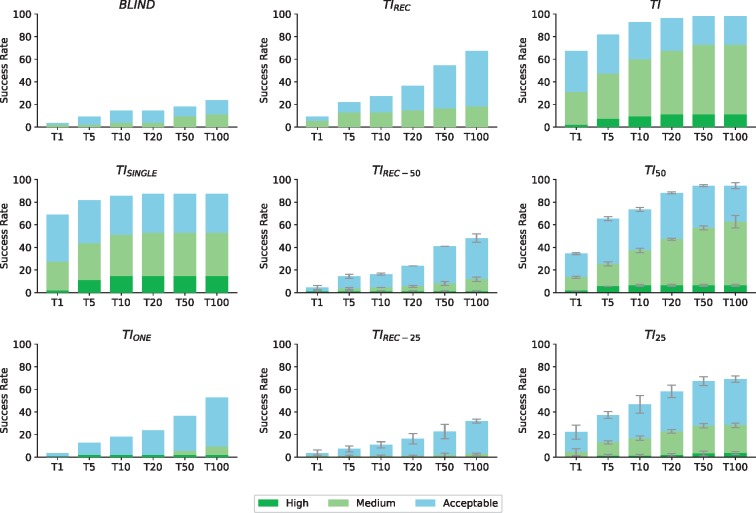
Performance of LightDock for the nine different scenarios. *BLIND*: *Ab initio* docking. *TI_REC_*: Only receptor contribution to the true interface. *TI*: All the residues from the true interface. *TI_SINGLE_*: A single residue pair from the true interface. *TI_REC-50_*: Half of the *TI_REC_* and equal number of non-interfacial residues. *TI_50_*: Half of the *TI* and equal number of non-interfacial residues. *TI_ONE_*: Only one residue on the receptor, as defined in *TI_SINGLE_*, is considered as restraint (i.e. no information on the ligand side). *TI_REC-25_*: One fourth of the *TI_REC_* and three times more non-interfacial residues. *TI_25_*: One fourth of the *TI* and three times more non-interfacial residues. True interface residues are calculated at a cutoff distance of 3.9 Å. Results are presented according to the CAPRI quality criteria (Lensink and Wodak, 2010) and the success rate is defined as the percentage of cases with at least one non-incorrect model within a given Top *N* (*N *=* *1, 5, 10, 20, 50, 100)

Figure 1 shows the results for the eight scenarios described above together with *ab initio* docking, which is included as a baseline for comparison purposes. The scoring function used in these LightDock simulations is DFIRE (Zhou and Zhou, 2009). When no prior information about the binding site is used for the docking calculations (*BLIND*), the predictive performance of LightDock lags behind any of the other scenarios tested in this work, with a moderate 14.5 and 23.6% success rates for Top10 and Top100 respectively. Interestingly, with the gradual use of information in the form of residue contact restraints, we find a boost in the performance up to a 92.7% for the Top10 when an accurate description of the interface (*TI*) is used. This represents an ideal case and illustrates how docking approaches can enormously benefit from integrating experimental data in their calculations. Unfortunately, structural experimental techniques rarely describe interfaces in a very accurate manner and the data produced is usually incomplete and/or incorrect, fact that heavily affect the performance of modelling approaches as previously discussed in Rodrigues and Bonvin (2014).

To account for inaccurate or incorrect data, we have designed artificial interfaces with false positive residues (Supplementary Material S4). When only 50% of the original *TI* is used (*TI_50_*) or 25% (*TI_25_*), which represents 50 and 75% of non-interfacial residues, LightDock performance in Top10 is of 72.7 and 46.4% respectively. In the case of *TI_50_*, Top100 performance compares to *TI* (94.6% versus 98.2%). This indicates that even when the information used to restrain the docking simulations in LightDock is incomplete and partially wrong, the protocol seems robust enough and still yields correct solutions for most of the cases (52 out of 55). However, the scoring becomes problematic compared to *TI* as the Top1 success rate drops from 65.5 to 33.6%.

In the scenario where only the contribution of the receptor is taken into account (*TI_REC_*), a substantial success rate of 67.3% is obtained for the Top100. This scenario is especially interesting since it directly applies, for example, to antibody-antigen docking where no information about the epitope is known so the docking is performed exploring the whole surface of the antigen while for the antibody the HV loops are provided (Supplementary Fig. S3). Moreover, when false positives are included in the *TI_REC_* scenario (50% in *TI_REC-50_*, 75% in *TI_REC-25_*) the performance drops, but Top100 is still higher (46.3 and 28.2%) than *BLIND* (23.6%).

Finally, we push the limits of the algorithm defining only one residue restraint on the receptor molecule (this would mimic one mutation data point for example). This effectively means that, as in *TI_SINGLE_*, only the ten closest swarms to the restraint will be generated, each of them containing randomly oriented glowworm poses (200 by default). In this scenario, restricting the sampling area helps the identification of near-native models as the performance is significantly higher than *BLIND* (Fig. 1). Remarkably, when we include a residue on the ligand molecule (*TI_SINGLE_*), which is used in the pre-orienting step (Supplementary Material S2), LightDock predicts and scores a near-native solution in the Top1 for 69% of the cases. From the different tested scenarios, it seems reasonable to state that our protocol enormously benefits from the additional data in form of residue restraints, even when it is incomplete and/or partially incorrect.

## 4 Conclusion

The new version of LightDock offers a powerful tool for modelling protein–protein complexes with high accuracy when good quality information about interfaces is available. Next to enabling the incorporation of data from mutagenesis and/or bioinformatics predictions, for example, this strategy might also be convenient in scenarios such as limiting the sampling to the solvent accessible loops of a transmembrane protein, or the CDR loops of an antibody. Moreover, when incorrect and/or incomplete data are used to restraint the simulation, LightDock is still robust enough to yield valuable predictions. While other FFT-based methods do support *a posteriori* filtering, the pre-filtering of swarms in LightDock does lead to a reduction of the computation time and a higher performance, which could be used to ensure a denser sampling around the binding region.

## Funding

This work was done with the financial support of the European Union Horizon 2020 projects BioExcel (675728, 823830) and EOSC-hub (777536) and of the Dutch Foundation for Scientific Research (NWO) (TOP-PUNT grant 718.015.001).

*Conflict of Interest*: none declared.

## Supplementary Material

btz642_Supplementary_Data
