# Supplementary material for: LightDock goes information-driven
Source: Bioinformatics. 2019 Aug 16;36(3):950–2. doi: 10.1093/bioinformatics/btz642 (PMC7005597; doi:10.1093/bioinformatics/btz642)
Supplement: btz642_Supplementary_Data [file btz642_supplementary_data.docx]

### Supplementary material for:

LightDock goes information-driven

Jorge Roel-Touris^1^, Alexandre M.J.J. Bonvin^1^ and Brian Jiménez-García^1*^

^1^Bijvoet Center for Biomolecular Research, Faculty of Science-Chemistry, Utrecht University, Utrecht, The Netherlands

*To whom correspondence should be addressed.

### S1. Swarms selection based on receptor residue restraints

LightDock simulations are organized in swarms over the receptor surface. Given an initial number of swarms *S* (by default 400) and residue restraints *R* specified by the user, we select the ten closest swarms to each residue in *R* (Euclidean distance). The set of swarms to be simulated is therefore the union of the different swarms selected for each restraint residue, which is a subset of the initial number of swarms *S*.

### S2. Glowworms pre-orientation based on ligand residue restraints

Each glowworm in the swarm encodes a given complex pose. The poses evolve in translational (Cartesian), rotational (Quaternions) and conformational space through an Anisotropic Network Model (ANM) space. The ANM model considers (by default) the ten first non-trivial normal modes calculated on the Ca and further extended to the rest of the atoms. These are included in each glowworm optimization vector to model backbone flexibility of both receptor and ligand molecules.

For each swarm, we select from the set of input restraints, the 10 closest receptor residues with respect to the geometric centre of the swarm ($R_{c}$). Then, we create random receptor-ligand restraint pairs $\{r, l\}$ where $r\in R_{c}$ and $l$ is a defined restraint residue of the ligand molecule. Finally, we orient each ligand pose using the vector facing the direction given by $\{r, l\}$. **Supplementary Figure 1** shows the preferred orientation of yellow arrows pointing towards the receptor restraint residues.

### S3. Score bias according to percentage of satisfied residue restraints

LightDock is somehow agnostic of the scoring function as previously discussed in (Jiménez-García *et al.*, 2018). The overall quality of the simulation will, of course, heavily depend on the capabilities of the selected scoring function to successfully describe the protein docking energetic landscape. In this new implementation, we calculate the intersection between the set of input restraints provided by the user and the set of those in contact for a given pose (3.9Å distance cutoff). The final score $\varepsilon_{f}$ (Eq. 1) of the complex is increased by the percentage of satisfied restraints (no penalties if none of the restraints is satisfied).

$\varepsilon_{f}=\varepsilon+P_{r}*\varepsilon+ P_{l}*\varepsilon$ (1)

$\varepsilon$ is the energy as calculated by the scoring function, and $P_{r}$ and $P_{l}$ are the percentage of satisfied restrained residues of the receptor and ligand respectively.

### S4. Design of artificial interfaces with the inclusion of false positive residues

For *TI_50_*, *TI_25_*, *TI_REC-50_* and *TI_REC-25_* experiments, we designed artificial interfaces, of equal size as our true interface description (*TI*), to include false positive residues in our restraint’s definition. For each of the cases, we clustered the *TI* residues in either 2 (*TI_50_* and *TI_REC-50_*) or 4 clusters (*TI_25_* and *TI_REC-25_*) of equal cluster size using the *AgglomerativeClustering* algorithm included in the *scikit-learn* python package (Pedregosa *et al.*, 2011).This algorithm recursively merges the pair of clusters that minimally increases a given linkage distance, for which we used Euclidean distance in order to assure contiguity between clustered residues in space.

At this point, we expanded the clusters (receptor and ligand separate interfaces) with the inclusion of the closest surface accessible (as calculated by ProDy (Bakan *et al.*, 2011)) neighboring residues at a maximum distance of 10Å, which were obviously not part of the *TI* definition. We stopped this expansion once the size (in terms of number of residues) of the generated artificial interface (true positive and false positive residues) is equal to our original *TI* description.

### Supplementary Figure 1: Representation of the filtered and pre-oriented swarms. Representation of two swarms (orange mesh) over the surface of a receptor protein (blue). In orange, the residues considered as restraints and therefore used to filter out the initial swarms prior the simulation. The initial orientations of the ligands within the swarms are represented using an orthogonal axis (x, y, z).

**
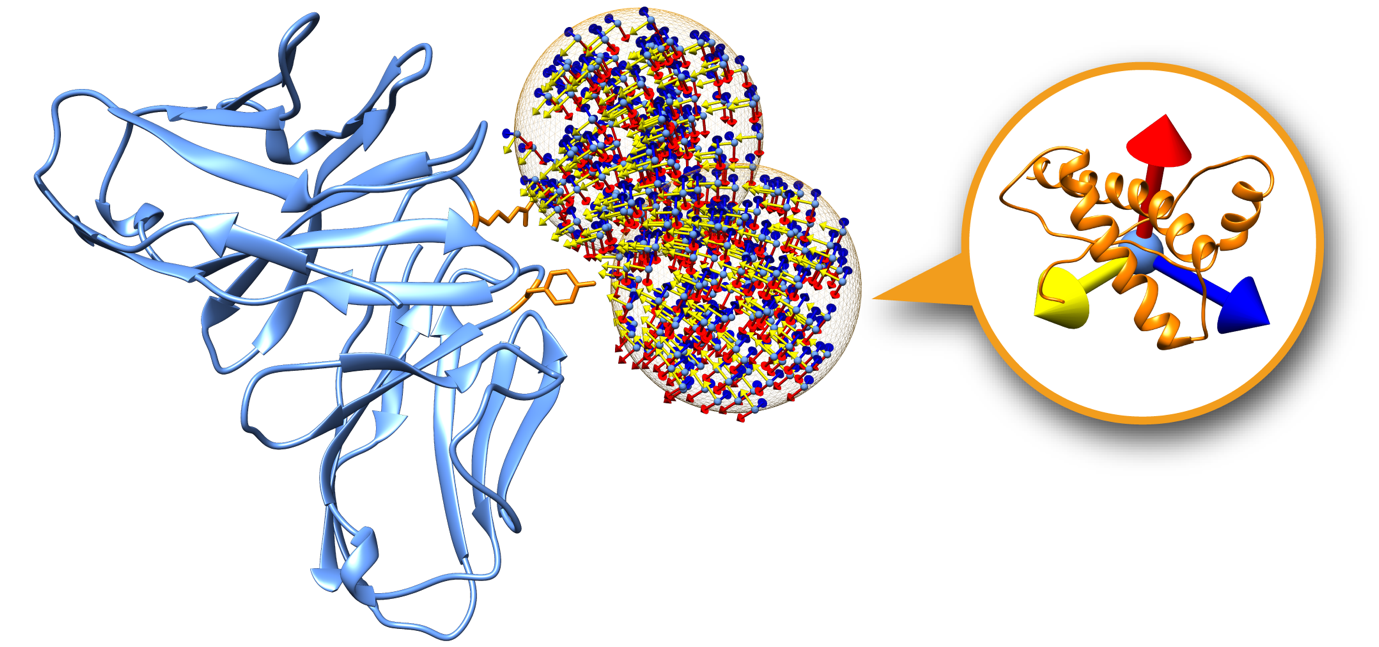
**

**Supplementary Figure 2: Comparison of performance of LightDock when data is used upon docking or as a post-filtering step.** BLIND: Ab-initio docking. BLIND-filtered: All the residues from the true interface are used as restraints for post-filtering BLIND predictions. TI: All the residues from the true interface used as restraints during docking. True interface residues are calculated at 3.9Å distance. The results are presented according to the CAPRI quality criteria (Lensink and Wodak, 2010) and the success rate is defined as the percentage of cases with at least one acceptable or higher quality model within a given Top N (N= 1, 5, 10, 20, 50 100).

**Supplementary Figure 3: Performance of LightDock for three different scenarios in a subset of 16 antibody-antigen complexes of the docking benchmark 5.** BLIND: Ab-initio docking. CDR: Only antibody CDR loops residues are considered as restraints (receptor). TI: All the residues from the true interface. True interface residues are calculated at 3.9Å distance. We defined the CDR loops as in (Ambrosetti *et al.*, 2019). The results are presented according to the CAPRI quality criteria (Lensink and Wodak, 2010) and the success rate is defined as the percentage of cases with at least one acceptable or higher quality model within a given Top N (N= 1, 5, 10, 20, 50 100). Results here presented represent an important increase of success rate compared to the ones in (Ambrosetti *et al.*, 2019), as current version (0.7.0) accounts for pre-orientation of ligand poses if information is available in contrast to version 0.5.6 where that information was not yet taken into account into the LightDock protocol.

### References

Ambrosetti,F. *et al.* (2019) Information-Driven Modelling of Antibody-Antigen Complexes. SSRN:https://ssrn.com/abstract=3362436

Bakan,A. *et al.* (2011) ProDy: Protein Dynamics Inferred from Theory and Experiments. *Bioinformatics*, **27**, 1575-1577.

Lensink,M.F. and Wodak,S.J. (2010) Docking and scoring protein interactions: CAPRI 2009. *Proteins Struct. Funct. Bioinforma*., **78**, 3073–3084.

Pedregosa,F. *et al*. (2011). Scikit-learn: Machine Learning in Python.

*Journal of Machine Learning Research*, **12**, 2825-2830
